# Cardiovascular risk factors differ between rural and urban Sweden: the 2009 Northern Sweden MONICA cohort

**DOI:** 10.1186/1471-2458-14-825

**Published:** 2014-08-09

**Authors:** Martin Lindroth, Robert Lundqvist, Mikael Lilja, Mats Eliasson

**Affiliations:** Department of Public Health and Clinical Medicine, Sunderby Research Unit, Umeå University, Umeå, Sweden; Department of Public Health and Clinical Medicine, Östersund Research Unit, Umeå University, Umeå, Sweden

**Keywords:** Risk factors, Cardiovascular, Urban, Rural, Cohort, Blood pressure, Anthropometry, Cholesterol

## Abstract

**Background:**

Rural communities have a higher burden of cardiovascular risk factors than urban communities. In Sweden, socioeconomic transition and urbanization have led to decreased populations in rural areas and changing characteristics of the remaining inhabitants. We investigated the risk factors in urban and rural populations in Northern Sweden.

**Methods:**

The 2009 Northern Sweden MONICA Study invited a random sample of 2,500 people, 25 to 74 years and 69.2% participated. Community size was classified as rural = <1,000 inhabitants, town = 1,000-15,000, or urban/city= >15,000. We adjusted our analysis for age, gender and education.

**Results:**

The rural population was older and the proportion of men was higher than in the urban areas. Having only primary education was more common in rural areas than in urban areas (26.2% vs. 12.3%). Waist and hip circumference, body mass index (BMI), and total cholesterol levels were higher in rural areas than in urban areas, even after adjusting for differences in age and gender. The largest differences between rural and urban dwellers were seen in waist circumference of women (4.8 cm), BMI of women (1.8 units) and cholesterol of men (0.37 mmol/l). Blood pressure was higher in rural areas, but not after adjusting for age and gender.

Participants in rural areas were more often treated for hypertension and hyperlipidaemia, hospitalized for myocardial infarction and diagnosed with diabetes. However, after adjusting for age and gender, there were no differences. The odds ratio for being physically active comparing rural areas to urban areas was 0.73 (95% CI 0.53; 1.01). Smoking, snuff use and the prevalence of pathological glucose tolerance did not differ between community sizes. Middle-sized communities often had values in between those found in rural and urban communities, but overall they were more similar to the rural population. Further adjustment for education did not change the results for any variable.

**Conclusions:**

In 2009 the rural population in northern Sweden was older, with less education, higher BMI, more sedentary lifestyle, and had higher cholesterol levels than the urban population. The rural population should be considered targets for focused preventive interventions, but with due consideration of the socioeconomic and cultural context.

## Background

In the USA and England cardiovascular disease (CVD) mortality is higher in rural regions than in urban regions [1, 2]. Data from Sweden are sparse, but official statistics show that regions dominated by rural communities have a higher incidence of myocardial infarction (MI) and stroke in both women and men [3]. Northern Sweden covers almost half of Sweden but contains only 1/9 of the population, thus combining a few large population centers by the coast with huge, sparsely populated areas in the inland. The incidence and mortality for MI and stroke have declined more rapidly in northern than in southern Sweden in the last decades, but there are still large geographical variations within the two northernmost counties of Norrbotten and Västerbotten [4].

In Västerbotten, public resources have been allocated to interventions against CVD risk factors through the Västerbotten Intervention Program (VIP), which has shown a decreasing burden of CVD risk factors over recent decades [5]. The Northern Sweden MONICA Study recently also reported large reductions in all risk factors except obesity and diabetes in Norrbotten and Västerbotten counties between 1986 and 2009 [6].

Although CVD is more prevalent and has higher mortality in the rural regions of Sweden, very little is known about the distribution of risk factors according to rural vs. urban living. An early study within the northern Sweden MONICA Study 1986 to 1999 showed that the burden of risk factors was higher in smaller communities [7]. Cholesterol, blood pressure, BMI and the prevalence of diabetes were higher, and low education was more common in communities with less than 1,000 inhabitants.

The VIP study found that those with only primary school education and who lived in rural, inland areas had consistently higher cholesterol levels and higher prevalence of hypertension than those living in urban areas and those with higher educational levels [8, 9]. Diabetes and obesity are more common in the sparsely populated regions all over Sweden [10–12].

Sweden has passed through great socioeconomic transitions during the last 30 years with a large migration from rural to urban areas, a flow that shows no sign of slowing down [10]. It is possible that those who stayed in the rural areas may have a less healthy lifestyle and therefore have higher levels of risk factors. Lower education in rural areas could enhance or mediate the risk, but causality is difficult to prove. Therefore, there is a risk that the differences between rural and urban areas in risk factors, incidence and mortality from CVD and diabetes will increase. Our aim was to describe and analyse differences between rural and urban populations in the 2009 MONICA population study, taking differences in age, gender and education level into account.

## Methods

We used data from the 2009 surveys of the WHO Northern Sweden MONICA study in Norr- and Västerbotten, the two northernmost counties of Sweden [6]. 2,500 randomly selected subjects between 25 and 74 years of age were invited from a target population of 312,000. The population was predominantly European, with only 2% being born outside Sweden or Finland, although ethnicity was not registered. Details of sampling, selection and measurement methods have been described previously [13].

Data of non-participants have been published [6]. Sixty two percent of the nonparticipants in answered a basic telephone questionnaire. Compared to participants, nonparticipants were on average younger, more likely to smoke, or report diabetes and less likely to have a university education or to be married/cohabiting. The use of antihypertensive medication or lipid-lowering drugs did or self reported BMI not differ.

Classification of rural, intermediate and urban living was derived from the survey question “Where do you live?”.

1. In a larger community, >15,000 inhabitants (coded as “city” or “urban”)
2. Other community with more than 1,000 inhabitants (coded as “town”)
3. Community with less than 1,000 inhabitants (coded as “rural area”)

Self-reported level of education was stratified into three groups based on the number of years in school, and these were defined as primary school secondary school and university or vocational training (>12 years of schooling). Blood pressure was measured twice in a sitting position, using a Hawksley random-zero sphygmomanometer, after a 5-min rest and the mean value was recorded.

BMI was stratified according to WHO criteria into normal (≤24.9), overweight (25–29.9) and obese (≥30). For adjusted values we used a dichotomized categorization with either normal (BMI < 25) or overweight/obesity (BMI ≥ 25). Blood samples for cholesterol measurement were drawn after a minimum 4-h fast and analyzed within 24 h without freezing; a dry chemistry method was used (Vitros 950; Kodak Ektachem, Rochester, NY, USA) [14].

A standard 75-g oral glucose tolerance test (OGTT) was performed in a random subset of participants, excluding patients with known diabetes. In total, 65% of all participants were invited to an OGTT. Venous plasma was used for glucose analysis [15]. Glucose tolerance was classified according to WHO, and pathological glucose tolerance was defined as impaired glucose tolerance (IGT) or diabetes.

Subjects who answered yes to the question “Do you have diabetes mellitus?” were classified as having ‘known diabetes’. Regular smokers were defined as those subjects who smoked at least one cigarette a day, all other were considered non-smokers. Participants reporting regular consumption of snuff were considered snuff users. Regular physical activity was classified according to the question “How much physical activity have you performed in the last year in your spare time?”. If the answer was “Hardly ever” or “Scarcely” the subject was classified as having a sedentary lifestyle. If the answer was at least 2 hr regular physical activity each week the individual was classified as physically active.

Previous cardiovascular disease was determined from the survey questions “Have you ever been hospitalized for a myocardial infarction?” and “Have you ever had a stroke?”. Participants were asked if they had taken prescribed medication against high cholesterol levels or hypertension within the last 2 weeks and classified accordingly.

MONICA is covered by multiple ethical permissions from The Regional Ethical Review Board, Umeå, Sweden, the latest in 2008. All participants gave written consent.

### Statistical methods

Mean values, standard deviations and proportions of risk factors are presented and compared between the cities, towns and rural areas using ANOVA and Chi-2 tests. Post-hoc tests adjusting for multiple comparisons with ANOVA were performed by Tukey HSD. The dependent variables were also modelled with univariate or logistic regression in order to adjust for age, gender and education. Odds ratios (OR) and 95% confidence intervals (CI) are presented. P-values below 0.05 were considered significant.

## Results

In 2009, a total of 2,500 subjects between 25 and 74 years of age were invited and 1,729 participated (69.2%). Data were missing in less than 5% for all variables. OGTT was only performed in 42% due to logistic reasons.

The mean age was higher in smaller communities (Table 1). All differences were statistically significant in pairwise comparisons between groups, by post hoc analysis with Tukey LSD test, except between women living in towns or rural areas. The gender distribution of the participants was unequal with men representing 55.9% of the rural population, 49.6% of the town population and 47.6% of the urban population (Table 2). Highest educational level differed among the different community sizes. Having only primary education was most common in rural areas, less common in towns, and even less common in rural areas.

**Table 1:** **Mean levels of cardiovascular risk factors according to community size**

		Age	SBP	DBP	Waist	Hip	BMI	Chol	fGlu	2 h-glu
**Men**	City	48.6 (14.7)	128.1 (16.0)	80.4 (10.5)	94.0 (11.0)	100.3 (6.7)	26.7 (3.8)	5.39 (1.1)	5.3 (0.7)	5.68 (2.1)
	Town	52.1 (12.6)	131.6 (18.3)	82.4 (10.3)	95.9 (11.5)	101.0 (7.3)	27.4 (4.5)	5.81 (1.2)	5.53 (1.3)	6.39 (2.7)
	Rural	56.7 (12.3)	131.8 (17.6)	82.3 (10.4)	96.8 (11.1)	101.2 (6.5)	27.5 (3.8)	5.76 1.2)	5.46 (0.7)	5.84 (2.2)
	*p*	<.001	0.009	0.031	0.007	0.2	0.018	<0.001	0.14	0.045
**Women**	City	48.3 (14.5)	122.6 (19.5)	76.2 (10.6)	83.1 (13.4)	101.1 (10.6)	26.1 (5.4)	5.44 (1.1)	5.30 (0.7)	6.45 (2.4)
	Town	52.4 (13.3)	125.5 (18.0)	77.1 (9.6)	86.0 (12.8)	102.4 (8.8)	26.9 (4.8)	5.77 (1.1)	5.26 (0.6)	6.56 (1.8)
	Rural	55.0 (11.9)	129.5 (18.7)	77.9 (9.0)	87.9 (13.9)	103.6 (11.2)	27.9 (5.9)	5.78 (1.3)	5.39 (0.7)	6.76 (2.3)
	*p*	<.001	0.001	0.16	<.001	0.027	0.001	<0.001	0.5	0.6
**All**	p*		0.3	0.16	0.006	0.013	0.001	0.013	0.6	0.6
	p**		0.4	0.14	0.014	0.024	0.004	0.016	0.7	0.4

Standard deviations within parenthesis. Analysis by ANOVA.

*SBP* = Systolic blood pressure, *DBP* = Diastolic blood pressure, Chol = Total cholesterol, *fGlu* = Fasting glucose, 2 h-glu = 2 hour glucose, p* = age and gender adjusted, p** = age, gender and education adjusted.

**Table 2:** **Proportions of cardiovascular risk factors and characteristics according to community size**

	City (%)	Town (%)	Rural (%)	***p***
**Male sex**	47.6	49.6	55.9	0.04
**Education level**				<.001
**Basic education**	12.3	18.3	26.2	
**Secondary school**	51.9	58.8	50.5	
**University**	35.8	22.9	23.4	
**Informed of hypertension**	29.3	33.0	39.6	0.003
**Antihypertensives last 2 wk**	16.7	19.5	27.2	<.001
**BMI category**				<.001
**Normal BMI**	45.1	35.0	31.0	
**Overweight** **(BMI** **>** **24.9** **<** **30)**	36.5	39.9	43.0	
**Obese** **(BMI** **>** **30)**	18.5	25.2	25.9	
**Cholesterol medicine**	9.7	14.1	17.4	<0.001
**Diabetes**	3.7	5.1	7.7	0.014
**Pathologic glucose**	19.5	21.6	25.8	0.3
**Regular smoker**	10.2	13.7	10.6	0.16
**Regular snuffer**	16.1	14.8	15.6	0.8
**Regular physical activity**	82.0	80.0	76.3	0.085
**Hospitalized for MI**	1.6	2.5	4.5	0.015
**Stroke previously**	2.6	2.5	4.4	0.2

Systolic blood pressure, in both men and women, was highest in rural areas and lowest in urban areas (Table 1). In a post-hoc analysis, men in towns and rural areas differed from those in cities while only women in rural areas differed from those in the cities. In men, diastolic blood pressure was higher in rural areas and small towns but not in women. In a post-hoc analysis, no differences were seen between any two groups and after adjusting for age and gender, no difference persisted in diastolic or systolic blood pressure. More participants had been informed that they had hypertension in rural areas than in towns and cities (Table 2). Treatment for hypertension was more common in rural areas, but after adjusting for age and gender no differences persisted.

Waist circumference differed between communities with the highest values in rural areas (Table 1) but in men only the difference between urban and rural areas was significant in post-hoc analysis. In women, waist circumference was higher in rural areas and towns compared to the cities.

Hip circumference did not differ among men but among women was higher in rural areas than in the cities in a post-hoc analysis. Differences in waist and hip circumference between different community sizes remained significant after adjusting for age and gender.

Inhabitants of rural areas had a higher BMI than those living in towns and urban areas but in men no differences were seen between any two groups (Table 1). In women, BMI was much higher in rural areas than in urban areas (p < 0.001 post-hoc Tukey HSD test). Differences in BMI across different living areas remained after adjusting for age and gender. In the rural areas more people were obese than in the urban areas (Table 2). Participants living in towns were similar to those in rural areas. Using the dichotomized categorization (Table 3), the odds ratio for overweight or obesity was 49% higher in rural populations than in urban areas, after adjusting for age and gender.

**Table 3:** **Categorical risk factors adjusted for age and gender**

	Town	Rural	***p***	p*
**Informed of hypertension**	0.98 (0.75: 1.28)	1.09 (0.82: 1.45)	0.8	0.7
**Antihypertensives last 2wk**	1.00 (0.72: 1.40)	1.22 (0.87: 1.71)	0.5	0.4
**Overweight/** **Obesity** **(BMI** ≥ **25)**	1.40 (1.09: 1.79)	1.49 (1.13: 1.98)	0.003	0.013
**Cholesterol medicine**	0.74 (0.51: 1.09)	0.83 (0.56: 1.24)	0.3	0.3
**Diabetes**	1.21 (0.69: 2.14)	1.44 (0.84: 2.49)	0.4	0.3
**Pathologic glucose**	1.05 (0.67: 1.64)	1.10 (0.68: 1.77)	0.9	1.0
**Regular smoker**	1.34 (0.94: 1.91)	0.95 (0.63: 1.46)	0.2	0.21
**Regular snuffer**	0.95 (0.67: 1.32)	1.01 (0.70: 1.46)	0.9	0.8
**Regular physical activity**	0.89 (0.66: 1.20)	0.73 (0.53: 1.01)	0.16	0.3
**Hospitalized for MI**	1.41 (0.61: 3.25)	1.65 (0.76: 3.55)	0.4	0.5
**Stroke previously**	0.83 (0.39: 1.78)	1.14 (0.57: 2.26)	0.8	0.7

Odds ratios and (95% confidence intervals). City is reference category (OR 1). p* = adjusted for age, gender and education.

Total cholesterol level was higher in the rural and town populations than in the cities in both sexes, but there were no differences between rural areas and towns (Table 1). Even after adjusting for age and gender the urban dwellers had lower cholesterol. Use of lipid-lowering agents was most common in rural areas and lowest in urban areas, but this was attributable to differences in age and gender (Table 2).

The mean fasting glucose level did not differ between communities of different sizes. By post-hoc analysis the two-hour glucose level for men was higher in towns than in cities and rural areas. No differences persisted after adjusting for age and gender (Table 1). Diabetes was most common in rural areas and decreased with increasing community size which was mainly explained by age and gender differences (Tables 2, 3). Prevalence of pathologic glucose tolerance did not differ (Table 2).

There were no differences in the prevalence of regular smokers or snuff users between communities of different sizes (Table 2). Regular leisure time physical activity tended to be most common in urban areas and least common in rural areas, but the difference was not significant (Table 2). Adjustment for age and sex further diminished this difference across groups but the odds ratio for being physically active comparing rural areas to urban areas was 0.73 (95% CI 0.53; 1.01) (Table 3).

Previous myocardial infarction was more common in smaller communities and lower in urban populations, mainly explained by differences in age and sex. The prevalence of previous stroke did not differ (Table 2).

Further adjustment of continuous variables for education, in addition to age and gender, showed that differences still existed for BMI, cholesterol, waist and hip circumference (Table 1). This was consistent with previous adjustments for only age and gender. For categorical variables, there were no changes in estimates after adding educational levels although the difference in physical activity between urban and rural areas was further diminished (Table 3).

## Discussion

In 2009, the rural population in northern Sweden was older and more often male, with less education, more abdominal obesity and higher cholesterol levels compared with the urban population. A sedentary lifestyle was more common in the rural areas. Thus, there is still a clustering of important cardiovascular risk factors in Swedish rural areas. Lack of jobs and opportunities to get an education influence young adults to move out of rural areas. A more diverse cultural life and a greater range of activities and entertainment attract younger people to urban living. Thus, it is probable that those who stay in the rural areas differ in many aspects from those who move to the urban environments.

The level of education differed between communities and the proportion of people with higher formal education was lower in rural areas than in towns and cities. Statistics Sweden reported a general increase in educational level among all Swedes including rural dwellers [16]. However, rural communities still have a higher proportion of elderly who grew up with only primary education since that was more common back in the 40s. As this generation subsequently will pass away, the proportion of individuals with only primary education will decrease in rural areas.

A low educational level has been linked to a shorter life expectancy [17] and to higher levels of cardiovascular risk factors [18]. A higher educational level might thereby improve the CVD risk factor profile of rural communities in the future, although a causal link has not been proven in prospective studies. Note that adjustment for education did not change any of our findings, thus adding further doubt to the hypothesis that low educational level is responsible for the more detrimental risk factor burden in rural areas.

Blood pressure and the proportion treated for hypertension did not differ. In the previous MONICA report from 1986–1999, the systolic blood pressure was higher in rural communities than in towns and urban areas [7], which was no longer evident in 2009. Possibly better awareness and treatment in rural areas has led to a decreased difference between rural and urban populations. From Västerbotten County, the VIP study reported an increased prevalence of hypertension in rural inland and coastland between 2005 and 2010 in contrast to the residential city of Umeå, which had a stable prevalence [8]. We analysed both Norrbotten and Västerbotten together, and there might be a discrepancy between VIP and MONICA since the trends may not be similar in the two adjacent counties, and the age ranges were not identical.

The prevalence of obesity and overweight were 50% higher in rural populations. A recent US report noted more obesity in rural settings, possibly causing higher risk of diabetes and coronary heart disease [19]. In northern Sweden no, or only slight, increases in diabetes have been found despite the increasing BMI [15, 20] and also CVD has decreased in both counties. However, it is possible that the rural population of northern Sweden might be at risk if BMI continues to increase. It should also be noted that obesity poses numerous other threats to health because obese people have a greater risk of sleep apnoea, stroke, cancer, osteoarthritis, gout, and back pain [3].

Waist and hip circumferences were higher in rural communities, which corroborates a previous Swedish study, although not population based [11]. Probable explanations are less favourable dietary habits and less amount of physical activity as compared with urban populations. The combination of waist and hip circumferences better predicts CVD mortality than waist circumference alone, which underestimates the risk [21, 22]. This is so, presumably by a better prediction of the amount of visceral fat. To that, an increased hip circumference might be beneficial both by potentially reflecting an increased muscle mass and by a protective physiology of the gluteofemoral adipose tissue [23]. The increased hip circumference seen in the rural population might therefore represent a metabolically beneficial sign, to some extent balancing the increased risk of co-morbidities linked to a disadvantageous increase in BMI and waist circumference. Still, a higher BMI and waist circumference in rural communities puts those persons at a higher risk of comorbidities associated with overweight and obesity.

We show that rural and middle-size communities have markedly higher cholesterol values (0.3-0.4 mm/L), but also that rural inhabitants use lipid lowering agents to a higher extent. Therefore we found no evidence for a treatment bias according to rurality, and those dwellers are not disadvantaged in regards to such medical care. On the other hand, it can be argued that with levels of total cholesterol that are still higher than in the urban areas, there is a need for more dietary changes and possibly also for more lipid lowering agents, at least among those at high cardiovascular risk.

High cholesterol levels have been noted in northern Sweden since the beginning of the WHO MONICA Project in 1986. This is regarded as the most important cause of a much higher CVD mortality in northern Sweden [4]. Data from MONICA show that cholesterol levels have decreased continuously between 1986 and 2009 [6], but in the VIP study there has been a shift in the positive trend after 2008–2010 [9]. This improvement in cholesterol has been seen in both rural and urban areas, but rural communities have had higher levels than urban areas throughout this period.

Much of the positive trend has been ascribed to public health interventions such as the Norsjö project, which evolved into the VIP Study [24], but it is possible that such interventions have been more successful in urban areas and perhaps better adopted by urban dwellers. Rural inhabitants have higher cholesterol levels mainly due to higher intake of saturated fat but also possibly due to more obesity, sedentary lifestyle and lower education [9, 12]. If cholesterol levels would reverse and increase in rural and middle-sized communities, it is possible that we would see an increased incidence and mortality from MI. The 2014 MONICA population survey will answer that question.

No significant differences based on community size in the prevalence of diabetes, fasting glucose or glucose tolerance were noted in our study. In a report from the northern Sweden MONICA between 1990–2009, an upward shift was seen in fasting and 2-h post-load plasma glucose levels, and increased prevalence of impaired glucose tolerance and impaired fasting glucose [15]. If this trend continues, the rural inhabitants might be afflicted to a greater extent since they have a higher proportion of obese individuals.

The use of tobacco in the form of moist oral snuff and smoking did not differ between communities in accordance with a previous study [7]. Regular leisure time physical activity was most common in urban areas and least common in rural areas. In a report from the Swedish Public Health Institute [25], a lack of studies from a rural perspective regarding physical activity in relation to the environment was observed. We know that a structural approach has been effective in urban areas with *i.e*. bicycle paths, walking trails in parks and good access to training facilities. Therefore, it is important to investigate further options to facilitate physically activity for rural inhabitants.

The development of society has made physically demanding employments, which were common in rural settings, very scarce today. Even forest and mining labour, which were previously very physically demanding, are now mostly sedentary and machine-operated by joy sticks. This needs to be compensated for by leisure time physical activity. In rural areas, long travelling distances, few walking and cycling trails and perhaps poor street lighting make motoring the traditional way to commute shop or visit friends. A structural approach is likely needed along with changes in culture or attitude to increase physical activity in rural settings.

In a report from the Swedish Public Health Institute 2007, health and it’s determinants in different types of municipalities were analysed [10]. The focus was on the structure of the municipality and the associated health of its inhabitants. Sparsely populated municipalities had more ill health markers than larger municipalities. Overweight, obesity, sedentary lifestyle and mortality from MI and diabetes were more common, and vegetable consumption was lower in rural areas than in large towns and similar municipalities. Age and educational attainment were the strongest predictors of ill health, but living in a sparsely populated municipality was still an explanatory factor for ill health after regression analyses had been performed. These findings corroborate our results and extend them to the whole of Sweden.

That report [10] also investigated what characterized the lifestyle of the inhabitants in sparsely populated areas. They found a higher proportion living under the social security or poverty level, higher incapacity rates and lower life expectancy in both women and men. On the positive side, inhabitants of sparsely populated regions reported better mental health and less stress.

Cultural aspects of rural living along with the socioeconomic situation might also affect how the rural population adapts to primary preventive advice from authorities. In a recent article the health of men and masculinity in rural areas of Sweden was discussed [26]. Structural factors, which characterized men in rural Sweden (versus urban Sweden) were lower educational attainment, lower-income jobs, and higher rates of unemployment. In rural communities a certain type of masculinity is encountered, such as hunting teams and snow scooter teams, which result in a lifestyle with less favorable eating, drinking and exercising habits. This male rural culture may also contribute to young women moving to cities more often than young men.

### Strengths and weaknesses

Similar to most modern population studies, participation rates declined over time in the MONICA study reaching only 69.2% in 2009. Non-participants differed from participants in some important aspects being, on average, younger and more likely to smoke or report diabetes. They were also less likely to have had a vocational or university education [6]. This may limit the external validity of the study and introduce some bias, especially among the youngest where participant rates were lowest. and caution is warranted in extrapolating the findings to the whole population. We do not know if participation rates differ by living area and therefore are not able to judge if comparisons between urban and rural living are affected.

The definition of urban and rural in our study was subjective. Small communities lying very close to a town or city might reflect a different way of living compared with small communities in very remote areas, which are common in northern Sweden. Participants may thus choose to report living in a rural setting even if they live very close to a city. Still, we show the expected differences in the distribution of cardiovascular risk factors in 2009.

Adjustment for educational level did not affect the results further after adjusting for age and gender. Since those with only primary education were probably represented mainly by the older population, the significance of education might be obscured by the higher mean age in rural communities. It is therefore possible that educational level might influence our results in a similar manner as age on the adjusted values of risk factors in our study. A high degree of collinearity between these two is evident.

BMI based on self-reported weight and length has been shown in many studies to be prone to underestimation, and the objectively measured BMI in MONICA adds further to the strength of this study.

## Conclusion

Most traditional cardiovascular risk factors differed between rural and urban communities of northern Sweden. When age and gender were taken into account, the difference in many of these variables was eliminated. Abdominal obesity and overweight were more prevalent, and cholesterol levels were higher, in rural communities even after multiple adjustments. A trend toward less leisure time physical activity was also found in rural communities. Thus, rural living in Northern Sweden is associated with higher levels of important risk factors. The rural population should be considered targets for focused preventive interventions, but with due consideration for the socioeconomic and cultural context. Otherwise we might face a surge in the incidence of CVD and diabetes in the rural populations.
